# Measuring Cortical Activity During Auditory Processing with Functional Near-Infrared Spectroscopy

**DOI:** 10.17430/1003278

**Published:** 2018-11-20

**Authors:** Luuk P. H. van de Rijt, Marc M. van Wanrooij, Ad. F. M. Snik, Emmanuel A. M. Mylanus, A. John van Opstal, Anja Roye

**Affiliations:** 1 Department of Otorhinolaryngology, Donders Institute for Brain, Cognition, and Behaviour, Radboud University Medical Center, Nijmegen, The Netherlands; 2 Department of Biophysics, Donders Institute for Brain, Cognition, and Behaviour, Radboud University, Nijmegen, The Netherlands

**Keywords:** near-infrared spectroscopy (NIRS), functional near-infrared spectroscopy (fNIRS), brain activity, auditory cortex

## Abstract

Functional near-infrared spectroscopy (fNIRS) is an optical, non-invasive neuroimaging technique that investigates human brain activity by calculating concentrations of oxy- and deoxyhemoglobin. The aim of this publication is to review the current state of the art as to how fNIRS has been used to study auditory function. We address temporal and spatial characteristics of the hemodynamic response to auditory stimulation as well as experimental factors that affect fNIRS data such as acoustic and stimulus-driven effects. The rising importance that fNIRS is generating in auditory neuroscience underlines the strong potential of the technology, and it seems likely that fNIRS will become a useful clinical tool.

## Background

Functional near-infrared spectroscopy (fNIRS) is an optical neuroimaging technique that assesses cerebral activity based on hemodynamics, which is associated with changes in the transmission of low power near-infrared light directed through the scalp and skull intothe brain (1). A variety of alternative terms have been used for the near-infrared spectroscopy (NIRS) technique, such as diffuse optical topography or tomography (DOT), diffuse optical imaging (DOI), and near infrared imaging (NIRI), although the underlying concept and physiological underpinnings remain similar (for detailed general reviews see e.g. (2–4)).

Brain activity leads to an increase in oxygen consumption, which is accompanied by an increase in cerebral blood flow due to neurovascular coupling (5). This induces a change in the local oxygenated (HbO_2_) and deoxygenated hemoglobin (HbR) concentrations. Given the different absorption coefficients of specific wavelengths of near-infrared light (600–900 nm) by HbO_2_ and HbR, changes in the concentration of each of these chromophores can be extracted by measuring changes in the amount of light transmitted over time (6). Due to the relatively low absorbance of near-infrared wavelengths by biological tissue, the cerebral cortex can thus be imaged. Specific parameters of the hemodynamic response observed with fNIRS hence reflect the spatial and temporal characteristics of changes in HbO_2_ and HbR, which may be manipulated by experimental paradigms and sensory stimuli (see below).

FNIRS is perfectly suited to the study of auditory processing in human subjects of all ages (7,8), since fNIRS is a non-invasive and silent brain-imaging technique, as opposed to PET (9) and fMRI (10). Further, the technique does not interfere with electromagnetic bionic devices such as cochlear implants (8,11). Since the technique is silent (as opposed to fMRI), subjects can be seated in a normal (laboratory) environment, in which they can readily perform real-world psychophysical tasks, and the technique can be easily coupled with simultaneous EEG recordings. Because of these advantages, an increasing number of researchers are seeing the potential of fNIRS in auditory research for both normal-hearing and hearing-impaired listeners (12).

The objective of this article is to review the current state of the art as to how fNIRS has been employed to evaluate auditory function, such as in speech, non-speech processing, and auditory attention in adults. In general, obtaining an optimal and stable setup and design for adequate hypothesis testing with fNIRS still remains a challenge. To test hypotheses of auditory processing requires a thorough understanding of the cortical hemodynamic response to acoustic stimuli, and how this response may be modulated by stimulus presentation rate, duration, sound level, and attention. Identifying the experimental factors that might affect the hemodynamic response is paramount for acquiring reliable and valid data.

The specific objectives of this paper are as follows: (i) to introduce the temporal and spatial characteristics of hemodynamic changes to auditory stimulation in general; (ii) to identify experimental factors that affect hemodynamic changes measured with fNIRS; (iii) to obtain insights into common experimental paradigms; and finally (iv) to summarize the contributions fNIRS has made so far to the study of auditory functioning.

## Temporal and spatial characteristics of the hemodynamic response

### Temporal characteristics of the hemodynamic response

FNIRS should be regarded as an indirect measure of neural activity, as it only measures vascular changes. The hemodynamic response to cortical neural activity relies on the fact that neuronal firing and the associated vascular response are strongly coupled (cf. neurovascular coupling; for a review see (13)).

**Figure 1:**
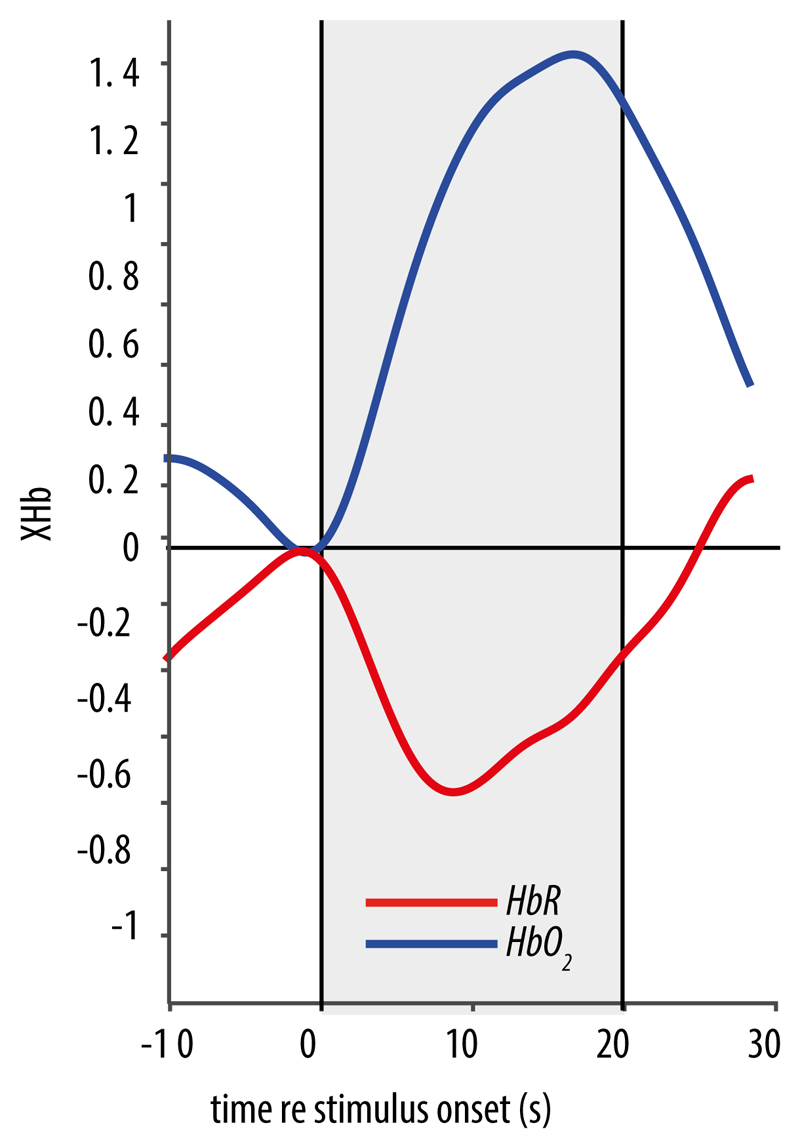
Hemodynamic response to auditory stimulation in temporal cortex. The blue line illustrates the increase of HbO_2_ and the red line the decrease HbR in response to the presentation of a speech stimulus (grey patch, 20 s). The sources and detectors were positioned over the left temporal hemisphere. Image adapted from Van de Rijt et al., 2016 (27).

Although crucial to this neuroimaging method, the mechanisms of neurovascular coupling are still not fully understood. It is clear that active neuronal tissue consumes energy for which the required inflow of oxygen and glucose will be accompanied by a local increase of cerebral blood flow, resulting in a local excess of oxygen in that particular area. This local increase of cerebral blood flow is associated with an increase of HbO_2_ and a decrease of HbR (see Figure 1 for an example). This characteristic behaviour is usually described as the hemodynamic response function (HRF), and is well characterized for adults (14,15). The characteristic HRF is related to the blood oxygenation level-dependent (BOLD) response that is also measured with fMRI (11,16), although the BOLD signal proper is assumed to reflect changes in HbR only (for a review on hemodynamic changes measured with fMRI see (17)).

In general, the onset of the hemodynamic response lags the much faster electrical neural response to sensory stimulation by about 2 s. The changes in HbO_2_ and HbR start with a steep increase, which rises to a plateau about 6–10 s after stimulus onset. The recovery time for the HbO_2_ and HbR responses to return to baseline is only infrequently reported (18), and is about 9–10 s (14). While both hemoglobin species (HbO_2_ and HbR) are well correlated regarding their temporal characteristics and shape during the steady state of the stimulus, sometimes an initial overshoot and a poststimulus undershoot may be observed for both chromophores (19). These are assumed to be a specific characteristic of neurovascular coupling (20).

Besides the general characteristics of the hemodynamic response, an important question is to what degree it is linearly related to the underlying neural activity, and hence whether it scales with stimulus input strength and obeys the superposition principle to multiple stimuli (on model linearity see e.g. (21)). For example, Soltysik et al. (23) reported that the auditory response obeys linearity for stimuli of a relatively long duration, but reveals nonlinear properties for short-duration stimuli (<10 s). It has also been suggested that responses become non-linear at higher stimulus presentation rates (22,24,25). Although, general aspects of hemodynamics might be partly responsible for non-linear response behaviour (e.g. saturation), another contribution could be due to the underlying neuronal responses, which can be enhanced by changes in the acoustic input, but will be suppressed for ongoing, tonic inputs (e.g., due to neural adaptation; for a review see (26)).

### Spatial information obtained with NIRS

**Figure 2:**
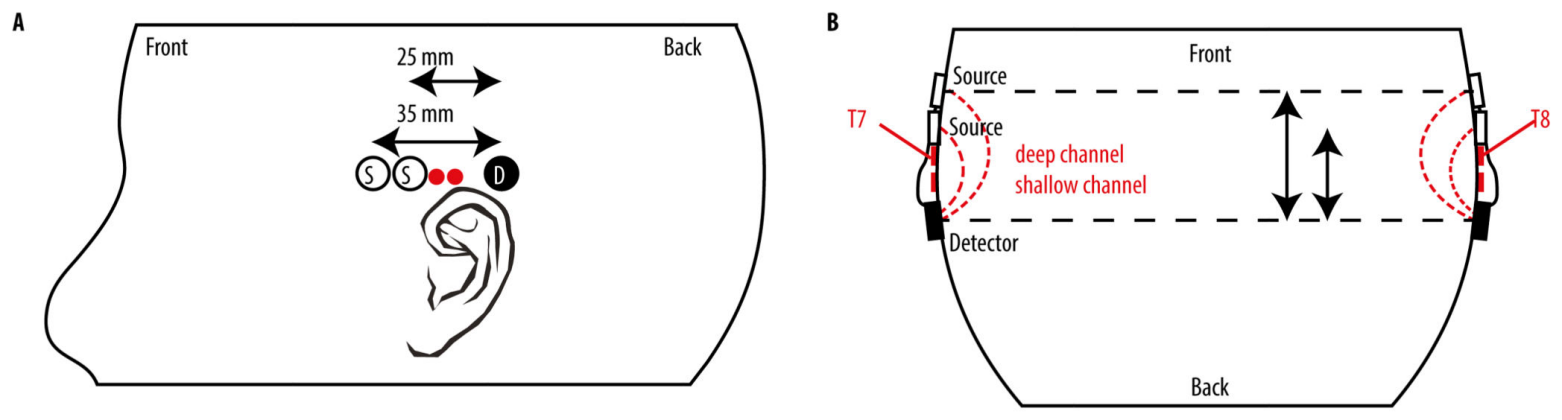
Positioning of the optodes. A) Layout of optical sources (open circles) and photodetectors (filled circles) on the left hemisphere; B) schematic top view of probe layout. The estimated T7 and T8 positions of a 10/20 system are also indicated, as these are the supposed superficial centers of the deep and shallow channels (red filled circles). Red dotted lines denote the average path from source to detector, estimated to be part of an ellipsoid with a penetration depth of approximately 2–3 cm. Image adapted from Van de Rijt et al., 2016 (27).

Figure 2 shows the probe template for two optical sources (S) and one photodetector (D) using source–detector distances of 25 and 35 mm, respectively (termed reference or shallow, and deep channel, respectively) (27). In this figure, the detector records the transmitted light coming from two sources, and each source–detector combination is defined as a channel. The sources transmit their light at unique frequencies in order to distinguish, using a lock-in amplifier, which source transmitted the light.

The first fNIRS measurements were carried out at only one or a few locations on the skull (11,28). Since stimulus-evoked brain activity occurs at restricted regions in the brain, one might miss the activation of interest when measuring just one brain area. Hence, a major step was to utilize multi-channel fNIRS systems which allow the possibility of measuring cortical hemodynamics from several cortical locations and construct topographic activity maps (29–32). Recently, researchers have developed a 140-channel fNIRS system to enhance local sensitivity – measured with several source–detector distances over overlapping regions to enable three-dimensional image reconstructions (33,34). The method resembles the topographic mapping techniques familiar with fMRI measurements.

b. Multi-channel measurements can certainly be regarded as an important development towards establishing fNIRS as a neuroimaging method that allows neuronal activity mapping over wide or distributed brain areas. However, unlike MRI, NIRS does not allow structural imaging of the brain, and so several refinements have to be made to overcome this limitation and allow reliable measurements and valid conclusions: 1) Positioning should be accurate and reproducible to guarantee that recordings are taken from the same location; 2) Valid inferences on targeted brain areas recorded with different channels should be possible. 3) One should remove systemic noise from cortical brain activity.

### Reliable positioning and valid inferences about underlying sources

Most researchers align the fNIRS channels (area between source and detector) with selected electrode positions of the well-established international 10-20 system (35–37). Although this procedure secures reliable positioning in general, conclusions about underlying cortical regions can only be drawn in a probabilistic manner (38). Obviously, some variance of the data will be attributable to the variability of defining the positions based on the 10-20 system across subjects and sessions (39).

Another option to enhance reliability and validity, and to avoid the variance induced by the 10-20 system, is to align recorded optode locations with anatomical positions of the channels by using magnetic resonance (MR) structural images. Investigators have used markers (e.g. alfacalcidol beads/ vitamin D or E) to determine which cortical structures were measured by fNIRS in the studied participant (40,41). This procedure ensures that the data were obtained from the region of interest, and therefore ‘auditory channels’ can be defined *a priori* (41,42).

A third way to improve reliability is by demonstrating spatial similarity in functional data obtained with alternative neuroimaging methods. Some research groups have used both fNIRS and fMRI to compare cortical measurements of speech-evoked activity (11). Others have used magneto-encephalography (MEG) and application of a 1000 Hz tone to determine the active region of the auditory cortex and so model the electric source of the N1m response (28).

Finally, implementing a localizer task into the experimental protocol of the fNIRS recordings itself, besides the experimental contrast, can also be a valuable method to determine regions of interest. For auditory experiments this could be a standard auditory stimulus (tones or noise), or the average response to all experimental stimuli used, which is then compared to a silent baseline period. Channels that exhibit maximal hemodynamic changes may then be followed up in further steps of the analysis (43). Alternatively, Kennan et al. [45] implemented a motor task (i.e. finger-tapping) within an auditory oddball task to localize the relative position of activation in primary motor cortex. These different approaches may contribute to improved inferences about target areas within and between studies.

### Distinguishing physiological noise from cortical brain signals

**Figure 3:**
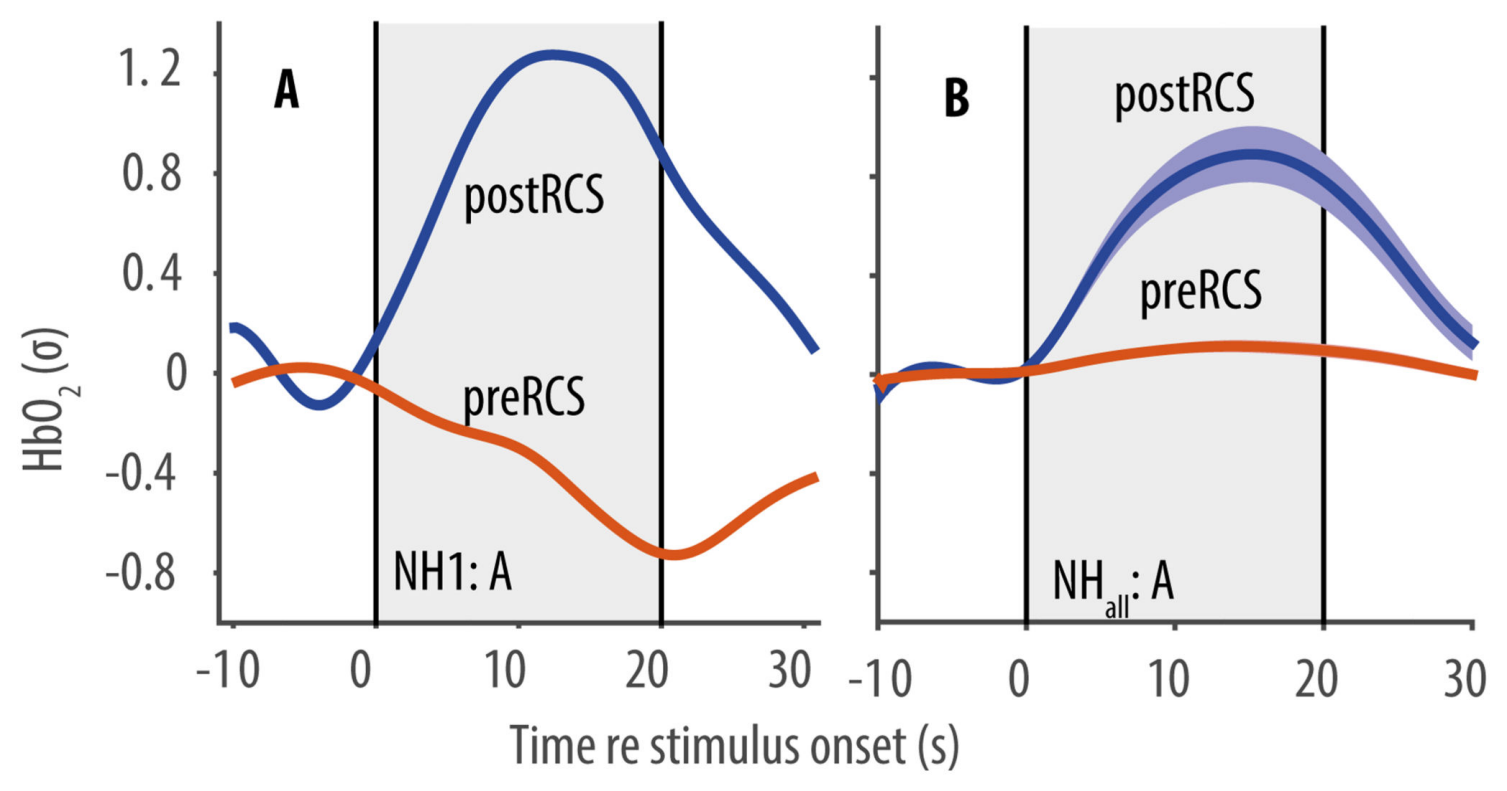
Reference channel subtraction. The red lines depict pre–reference-channel subtraction and the blue lines depict post–reference-channel subtraction. Grey patches indicate auditory stimulus presentation. Stimulus presentation was 20 s. A) Averaged normalized HbO_2_ data for 12 auditory stimuli of a normal-hearing subject (NH1); B) the same for a normal-hearing cohort (n = 33). Image adapted from Van de Rijt et al., 2016 (27).

When looking at the raw fNIRS signals recorded from NIRS channels, which are supposed to target certain brain areas, systemic or physiological noise often pollutes the hemodynamic responses of interest. These physiological sources of noise, such as heart beat, respiration, or Mayer waves (44) may hide experimental effects which are usually of much smaller amplitude, and it may require sophisticated methods to identify the latter. Using a ‘reference channel’ offers a possible way to increase the reliability of estimating the hemodynamic response from fNIRS signals (27,44,45). A reference channel is characterized by a short source–detector distance (range of 1–2 cm, see Figure 2), and makes use of the direct relation between source–detector distance and depth reached by photons in tissues underlying the scalp (46–48). Due to the short distance of the reference channel, it is likely to reflect hemodynamic activity that is taking place within superficial tissues rather than stimulus-evoked brain activity. Signals derived from the reference channel seem to be perfectly suited for subtraction of physiological noise from the measured NIRS signal (i.e. reference channel subtraction (RCS)), and has been demonstrated to facilitate the estimation of evoked cortical hemodynamic responses (49–51) (Figure 2). In Figure 3 an example is shown of how, at the single-subject level, RCS affects the average response during auditory stimulation. In general, it improves the signal response (Figure 3B; for further explanation see (27)).

### Choosing the most appropriate experimental paradigm

Besides potential methodological difficulties in placing the optodes and removing physiological noise, a further important consideration for optimising data quality is the experimental paradigm used in an fNIRS study. With a few exceptions (37,52), the majority of NIRS studies employ a block design. In this approach, the different experimental conditions are presented separately within relatively long blocks (4–30 s) of stimulation. Within each block, tokens of the same stimulus type are presented repetitively, or in an ongoing manner. Stimulation blocks are followed by a control condition to allow for the HRF to return to baseline. These periods are usually filled with silence or some kind of unrelated stimulation during the rest period to reduce movement artefacts and keep participants attentive to the experiment (11).

The general benefit of a block design is reflected in the robustness of the obtained hemodynamic signal. Due to repetitive presentation of a stimulus condition within a block, the captured HRF of the entire block is acquired as a superposition of the individual HRFs to each stimulus presentation. However, this design also has its shortcomings. The effects of individual stimuli within a block cannot be obtained (e.g. different responses to different words within sentences). Further, due to relatively long blocks of stimulation, the obtained responses might be influenced by effects of arousal, selective attention, or other cognitive effects that may vary between blocks and hence confound the actual effect of interest.

As an alternative to the block design, an event-related design (37,52,53) can overcome these attention- or task-related effects. In this case, relatively short stimuli (1–4 s) are presented in much faster succession than the different blocks in a block paradigm. Faster stimulation reduces data acquisition time and hence the total number of epochs (events) can be increased compared to the block design. For the design of the experiment, it is important to consider that the time between two successive stimuli can be short, but should be long enough to allow the HRF to partially return to baseline in order to avoid saturation of the hemodynamic signal. Jittering the inter-stimulus interval may also contribute to reducing random physiological noise in the data. However, due to overlapping HRFs, statistical analysis of the data requires more sophisticated approaches than does a block paradigm (e.g. a general linear model (GLM), see (54); for a review, see (55)).

## Modulating the hemodynamic response by experimental variations

### Stimulus-specific and area-specific activations

While NIRS may be considered a reliable and valid tool to study stimulus-driven, bottom-up visual processing (56), clear evidence that NIRS reflects stimulus-specific and modality-specific activations to acoustic stimuli still needs to be established. The lack of clear evidence is partially due to the use of only a limited number of optodes, and hence *a priori* areas of interest, but also to a lack of systematic experimental designs that target modality and stimulus specificity. The first limitation is overcome by using multi-channel fNIRS that allows spatial brain mapping. It has been shown that maximal hemodynamic changes are indeed measured when channels are centered on the auditory cortex, whereas the optical signal diminishes or disappears for locations away from auditory cortex (30,43,57,58). This regional specificity of activations is further supported by studies which have demonstrated differential activations at the expected occipital (V1), auditory (A1), and sensorimotor cortical regions for visual stimuli (e.g. checkerboard stimulation), motor tasks (e.g. finger-tapping), and auditory stimulation (tones), respectively (43,59). More specifically, a recent study by Chen et al. (59), which measured auditory and visual areas in response to stimuli of both modalities, appeared capable of dissociating auditory from visual activations by showing maximal responses in the associated modality-specific areas. Prior to this certainly necessary systematic experiment, several prior studies had already demonstrated that the hemodynamic response to auditory stimuli can be altered by varying basic, as well as higher level, sound characteristics (bottom-up effects), and also by including top-down task characteristics within the same modality.

### Acoustic stimulus driven effects on the hemodynamic response

#### Loudness modulation

Most studies performed with fMRI have demonstrated that the auditory hemodynamic response is sensitive to variations in sound level (60,61). Some authors have indicated a positive, nearly linear relationship between the strength of the BOLD signal and sound intensity (62). It appears that auditory cortical responses measured with fNIRS show such a linear relationship for *perceived* loudness, rather than for the (physical) intensity of the sound (59). This potential discrepancy between intensity vs. loudness might suggest that fNIRS does not primarily target primary auditory cortex, where intensity effects seem more clear, but mainly relate to activity generated in secondary auditory areas (see discussion in (59); also on fMRI (63,64)).

#### Presentation and repetition rate modulation

A difference between block and event-related designs is the interval between consecutive stimuli, which is longer for a block design (3–25 s) and relatively short in an event-related design (1–4 s). When experiments discuss the interval between two stimuli, a clear distinction needs to be made as to whether one is referring to the inter-stimulus interval (ISI) between consecutive stimuli (usually referred to as the presentation rate) or to the inter-stimulus interval between identical stimuli (called the repetition rate). Generally, most studies indicate a nonlinear, inverse relationship between the cortical response and the stimulus presentation rate. As stimuli are presented in fast succession, the cortical response reaches a plateau and may even decrease (evidence from fMRI, (24,65–68); see also section on the temporal characteristics above). With fNIRS, the effect of sound presentation rate on cortical activation has been investigated by Weiss et al. (69). These authors systematically looked at presentation rates of trains of noise bursts at 2, 10, and 35 Hz. The study confirmed an inverse relationship between HbR concentration change and presentation rate.

In addition, there is the phenomenon of stimulus-specific neural adaptation (for a review see (70)), which holds that responses to an immediately following stimulus (i.e., at short ISI) can be influenced by the response to an immediately preceding stimulus. The size of the response will be reduced if specific stimulus characteristics are repeated. That is why it is useful to distinguish the presentation rate (which concerns different stimuli) and the repetition rate (which refers to identical stimuli). If sufficient time has elapsed before the *same* stimulus is repeated, suppression of the hemodynamic response to the latter may be absent.

A nice illustration of this phenomenon is the ‘oddball paradigm’ (see e.g. (52). In a standard oddball paradigm, the subject is presented with a series of repetitive or ‘standard’ stimuli that are randomly and infrequently replaced with a distinctly different or ‘deviant’ stimulus. When an identical stimulus (usually called the standard stimulus) is presented several times, the neural system will adapt, leading to reduced neuronal activity for the consecutive stimuli. As a result, the hemodynamic response may saturate (71). This has been shown by Kennan et al. (52), who used a classical auditory oddball design. In the same way, continuous tones do not produce an ongoing hemodynamic response. However, their study also showed that even if the presentation rate is quite high and hence the ISI is short (1.5 s), a low repetition rate of the rare stimuli (which deviate from the repeated standard stimulus) can result in clear responses to these stimuli, even when presented within generally fast sequences of other stimuli. By observing these responses, it makes the technique suitable for experiments which do not last long (e.g. for children).

#### Stimulus complexity and impact of higher order stimulus categories

Based on fMRI, PET, and animal studies, it can be hypothesized that acoustic complexity can modulate hemodynamic responses. Simple acoustic stimuli (e.g., pure tones) primarily activate the core of the primary auditory cortex, whereas spectrally more complex sounds (e.g., complex noise, vocalizations, music, speech) also activate the surrounding higher order areas (e.g. (72,73); for a review see (74)). So far, only one study used both simple tones and more complex frequency-modulated sounds within the same fNIRS study (59).

Besides acoustically driven effects, some research groups have also investigated whether fNIRS shows sensitivity to higher order stimulus features. For example, Pollonini et al. [34] varied intelligibility of auditory stimuli using sounds with otherwise comparable acoustic features (frequency content, spectro-temporal modulations, intensity). They showed that meaningful and intelligible auditory inputs led to a broader area of activation within temporal cortices. The activation decreased for distorted sounds or for non-speech environmental sounds. Bembich et al. reported fNIRS activation only for meaningful words, when compared to meaningless vowel-consonant-vowel syllables (36). Further, several studies by Minagawa-Kwai et al. (30,57,58) suggested that fNIRS is sensitive to language-specific speech contrasts. They demonstrated that there were left hemispheric hemoglobin changes to phoneme contrasts within the listener’s native language that was not present for phoneme contrasts measured in non-native listeners. This left side functional lateralization seems to be driven by the phonemic contrast of the speech, since Sato and colleagues (42) demonstrated that a *prosodic* contrast led to right-sided dominance.

That the emotional valence of non-speech sounds can also yield differences in hemoglobin changes has been shown by Plichta et al. (43), who reported that both pleasant and unpleasant sounds led to significantly enhanced hemoglobin changes in auditory cortex when compared to neutral sounds. Another group looked into the effects of fear and disgust (75), and showed that sounds that were associated with fear elicited increased hemoglobin changes within temporal–parietal regions, while disgusting sounds elicited smaller changes. Taken together, these findings underscore that internal representations such as language-specific experiences, and emotional or motivational relevance, can lead to hemoglobin changes that are measurable with fNIRS.

### Top-down effects on the hemodynamic response

As described above, auditory cortical responses measured with fNIRS depend on many stimulus-driven factors such as presentation rate, loudness, complexity, intelligibility, experience, and emotional valence. Only a few studies have systematically looked into the effects and response dependencies of attention and task-demands, although it has been suggested by other recording methods that the attentional focus can influence auditory cortical responses (for a meta-analysis on fMRI data see e.g. (76); for a general review see (77)). Often, fNIRS studies do not really control for attentional effects and simply require the subject to listen without giving a certain response (11,28,75,78). A notable exception is the fNIRS study of Kojima and Suzuki (79), which utilized visual stimuli to show that hemodynamic responses in visual cortex are enhanced when participants are asked to perform a visual search task (compared to the inattentional condition).

For auditory stimulation the fNIRS study of Remijn and Kojima (80) assessed auditory-cortical responses within a streaming paradigm. Their results showed that performing a task of actively judging a perceived acoustic rhythm caused significantly larger HbO_2_ responses compared to the passive listening condition. In summary, several studies suggest that hemodynamic responses driven by auditory stimulation can be enhanced through auditory attentional engagement.

## Reproducibility of fNIRS measurements

A potential advantage of the NIRS technique, compared to other neuroimaging methods, is that the brain activity of patients wearing hearing aids or implants, and also of children, may be measured in a clinical setting. However, a prerequisite for using the technique is to assess its general *reproducibility* or retest reliability. To our knowledge, no study has formally evaluated the reproducibility of different aspects (size, location, amplitude, temporal behaviour) of hemodynamic responses elicited by auditory stimulation. For other modalities, some multichannel fNIRS studies have been carried out to evaluate retest reliability (in the motor cortex, see (39,81); in the occipital cortex to visual stimulation, see (53)) and they suggest that reliability at the group level exists.

So far, two studies have looked at the reliability of cortical activation in an event-related design (53,81), while Sato et al. (39) has looked into data reproducibility using a block design. The authors demonstrated that absolute signal amplitudes may vary between sessions, but that the time courses of the signal are highly correlated between sessions (*r* > 0.8). To address the level of reproducibility of fNIRS in occipital cortex, Plichta et al. (53) presented periodic checkerboard stimuli and measured them at a retest interval of 3 weeks, focusing on three different aspects. First, the reproducibility of a number of activated channels over the two sessions was moderate. Second, in a single channel comparison the reproducibility was generally low, but this improved when channels were clustered (significant activations at first and second session). As a last step, they looked at topographic map activation (*t*-values) within their pre-defined region of interest, and this showed that the fNIRS group activation maps were highly reproducible.

These outcomes show that, on a group level, fNIRS is reliable and trustworthy for fundamental research looking into effects on subjects. However, at this point, reproducibility in single subjects seems to be lacking (53,81–83). Different causes may underlie this problem. As mentioned before, often only a very limited set of fNIRS optodes is measured, and even if the researcher increases their number, makes exact and reliable positioning, uses data-driven channel selection, and analyses signals over broader areas of interest, these refinements do not always reduce between- and within-subject variance. Some authors suggest implementing MRI-guided techniques (84) to improve within-subject reliability. However, since fNIRS is intended to be used on subjects for whom fMRI scans are to be avoided (children, auditory research, participants with bionic devices), the alignment of fNIRS outcomes with structural and/or functional MRI scans is not an ideal solution.

## Conclusions

This review has aimed to summarize the state of the art of how fNIRS can be used to study auditory central processing. This review indicates that increasing numbers of auditory neuroscience researchers are now readily using fNIRS to measure hemodynamic responses to a range of experimental stimuli and response conditions. Yet, despite the promising results of fNIRS, developing an ideal and stable setup and experimental design for adequate hypothesis testing still remains a challenge. By incorporating some of the aspects reviewed here – for example, details of how the cortical hemodynamic response to acoustic stimuli is modulated by stimulus presentation and repetition rates, sound duration, sound level, and attention – one might be able to acquire reliable and valid fNIRS data.

For further details on the underlying physiological principles (85,86), available analysis methods, and technological advancements in fNIRS (aspects which lie outside the scope of this review), we suggest reading existing reviews (2,3,55).

An important asset of fNIRS is that it can be readily combined with other neuroimaging modalities such as fMRI, EEG, PET, and MEG. Evidence comes from the increasing number of publications on multimodal imaging systems (28,37,52,87,88).

FNIRS is becoming increasingly recognised as a powerful neuroimaging tool to reveal cortical activity in different patient groups of all ages. Typically, this neuroimaging method is silent and non-invasive, as opposed to fMRI and PET respectively. Furthermore, the technique is not impeded by electromagnetic bionic devices, such as a cochlear implant (CI). Anderson et al. (12) has recently shown the potential importance of applying fNIRS for longitudinal studies of cortical auditory function in CI users, giving insights into the correlation between audio-visual interactions and cortical reorganization, before and after cochlear implantation. Their results provide evidence of cortical plasticity within the bilateral superior temporal cortex (STC), suggesting how these effects may potentially explain the considerable variability in CI outcome measures.

## List of abbreviations

BOLD: blood oxygenation level dependent
CI: cochlear implant
DOT: diffuse optical tomography/topography
fNIRS: functional near-infrared spectroscopy
fMRI: functional magnetic resonance imaging
GLM: general linear model
HbO_2_: oxygenated haemoglobin
HbR: deoxygenated haemoglobin
HRF: hemodynamic response function
ISI: inter-stimulus interval
MEG: magneto-encephalography
MR: magnetic resonance
MRI: magnetic resonance imaging
NIRI: near-infrared imaging
NIRS: near-infrared spectroscopy
PET: positron emission tomography
RCS: reference channel subtraction
STC: superior temporal cortex
